# Social Support, Self‐Management Behaviors, and Coping Styles of Patients With Wilson's Disease: A Quantitative Empirical Research

**DOI:** 10.1002/brb3.71138

**Published:** 2025-12-22

**Authors:** TingTing Zhan, Xiang Li, Yan Wang, Lili Wang, Caijie Sun, Xiaohan Hu, Shutong Qiao, Xue Li, Wenjie Tu

**Affiliations:** ^1^ Department of Neurology The First Affiliated Hospital of Anhui University of Traditional Chinese Medicine Hefei Anhui Province China; ^2^ Graduate School of Nursing Anhui University of Traditional Chinese Medicine Hefei China

**Keywords:** coping style, Roy's Adaptation Model, self‐management, social support, Wilson's disease

## Abstract

**Purpose:**

Based on Roy's Adaptation Model, this study explores the relationship between social support, coping styles, and self‐management behaviors in patients with Wilson's disease (WD), as well as the underlying mechanisms.

**Methods:**

A cross‐sectional survey was conducted among 230 WD patients from a tertiary hospital using the Social Support Rating Scale, the Simplified Coping Style Questionnaire, and the Chronic Disease Self‐Management Study Scale. Structural equation modeling was employed to analyze the pathways between variables.

**Results:**

The total scores for social support, coping styles, and self‐management behaviors were 36.03 ± 7.71, 50.44 ± 10.95, and 37.76 ± 12.05, respectively. Social support was positively correlated with self‐management behaviors (*r* = 0.386) and positive coping (*r* = 0.578) and weakly correlated with negative coping (*r* = −0.170). Positive coping was positively correlated with self‐management behaviors (*r* = 0.519), while negative coping was negatively correlated (*r* = −0.308). Structural equation modeling indicated that coping style partially mediates the relationship between social support and self‐management (*β* = 0.199, *p* < 0.001).

**Conclusion:**

Social support directly enhances self‐management behaviors in patients with WD and indirectly promotes them by fostering positive coping styles, supporting Roy's adaptation model as a theoretical basis for intervention development.

**Implications for Practice:**

Effective self‐management in WD patients requires a combination of factors, including the use of social resources, reducing negative perceptions of the disease, and adopting positive coping strategies.

**Impact:**

Based on Roy's adaptation model, to explore the self‐management behaviors of patients with hepatolenticular degeneration and their influencing factors. We identified the correlation between self‐management behaviors and social support and coping styles in patients with Wilson's disease. This study provides a theoretical basis for improving the self‐management behaviors of patients with Wilson's disease and lays a foundation for further empirical research.

## Abstract 2

Based on the Loy adaptation model, this study explored the relationship and intrinsic mechanism among social support, coping styles, and self‐management behaviors in patients with hepatolenticular degeneration (Wilson's disease or WD). A cross‐sectional survey was conducted among 230 WD patients in a tertiary hospital using the Social Support Rating Scale, the Simple Coping Style Questionnaire, and the Chronic Disease Self‐Management Research Scale. And use the structural equation model to analyze the path relationship between variables.

## 1 Introduction

Wilson's disease (WD), also known as hepatolenticular degeneration, is a rare autosomal recessive genetic disorder (Karantzoulis et al. 2021). Patients with WD exhibit diverse phenotypes, typically presenting with hepatic and/or neurological symptoms. The age of onset ranges from 8 months to 74 years (Abuduxikuer et al. 2015; Ala et al. 2007; Członkowska et al. 2008). Effective management of WD is grounded in early diagnosis and lifelong treatment. Management requires lifelong pharmacological therapy and adherence to a low‐copper diet, both of which demand strong self‐management skills (Pellecchia et al. 2003). Self‐management, based on social cognitive and self‐efficacy theories, emphasizes the patient's ability to effectively regulate and maintain their health condition. However, research on self‐management in patients with WD remains scarce, particularly studies guided by systematic theoretical frameworks.

Roy's Adaptation Model emphasizes that individuals respond to internal and external stimuli in four adaptive modes—physiological, self‐concept, role function, and interdependence—to maintain or restore adaptation (Roy 2009). This model offers a systematic perspective to understanding health behaviors in chronic disease patients. Social support, as an external psychosocial resource, plays a crucial role in protecting mental and physical health, alleviating stress, and reducing psychological distress (Folayan et al. 2020). Coping style is another key factor influencing self‐management, reflecting cognitive and behavioral strategies within the self‐concept mode (Bandura 2001). Positive coping helps enhance treatment confidence, alleviates negative emotions, improves mental well‐being, and strengthens self‐management capabilities (Zaki et al. 2021). Although the psychosocial aspects of Wilson's disease are gaining recognition, empirical research on the mechanisms underlying self‐management remains limited. Recent international studies, such as the work by Göktaş and Yalcin (2024) in pediatric WD, highlight the global challenge of treatment adherence. Complementing this evidence, the present study focused on adult patients and aimed to quantitatively investigate the roles of social support and coping styles as key psychosocial determinants of self‐management, thereby expanding the current understanding of this condition from a distinct perspective.

Given the limited research on self‐management in WD patients, this study aimed to: construct a relationship model linking coping style, social support, and self‐management based on Roy's Adaptation Model. Explore the impact of coping style and social support on self‐management behaviors; Provide a theoretical foundation for designing targeted nursing interventions to improve self‐management behaviors and enhance adaptation in WD patients.

## 2 Materials and Methods

### 2.1 Ethical Considerations

This study was conducted in accordance with the Declaration of Helsinki and approved by the Ethics Committee of the First Affiliated Hospital of Anhui University of Chinese Medicine (Approval No: 2021AH‐06; Date: October 6, 2021). All participants provided written informed consent.

### 2.2 Theoretical Framework

Guided by Roy's Adaptation Model, social support was conceptualized as an external contextual factor influencing the interdependence mode, coping style as a core component of the self‐concept mode, and self‐management behavior as an adaptive response within the physiological and role function modes.

### 2.3 Sampling and Recruitment

A convenience sampling method was employed to recruit WD inpatients from the Encephalopathy Center of the First Affiliated Hospital of Anhui University of Chinese Medicine—the largest WD research center in Asia. Before administering the questionnaire, all participants were informed of the study purpose and procedures.

### 2.4 Sample Size Calculation

According to descriptive research guidelines, the sample size should be at least 5–10 times the number of variables (Östlund et al. 2011). This study included eight dimensions across three scales assessing social support, coping style, and self‐management behaviors, as well as 10 dimensions assessing general data such as age, sex, and duration of illness—resulting in a total of 18 dimensions. Considering the potential for invalid questionnaires due to incomplete or low‐quality responses, the sample size was increased by 20%. Accordingly, the minimum and maximum required sample sizes were calculated as follows:

$$N_{\min}=\left(18\times 5\right)\times \left(1+20\%\right)=108$$

$$N_{\max}=\left(18\times 10\right)\times \left(1+20\%\right)=216$$

During data collection, several partner institutions proactively contributed additional eligible cases. Ultimately, 230 questionnaires were distributed, all of which were returned with valid responses.

### 2.5 Inclusion and Exclusion Criteria

Patients were included if they met the following criteria: aged ≥18 years; diagnosed according to the Chinese Guidelines for Diagnosis and Treatment of WD (Wu et al.,^2021^); without communication barriers; able to complete the questionnaire independently or with assistance. Patients with comorbid conditions that could impair their ability to complete the survey were excluded.

### 2.6 Instruments and Their Validity/Reliability

A cross‐sectional survey was conducted using data from May 2022 to August 2022, and 230 WD patients who were treated in a tertiary hospital in Anhui Province completed it. Data were collected using the following instruments: A self‐designed general information questionnaire; the Simplified Coping Style Questionnaire (SCSQ; Cronbach's α: 0.78–0.89); the Social Support Rating Scale (SSRS; Cronbach's α: 0.90); the Chronic Disease Self‐Management Study Scale (CDSMS; Cronbach's α: 0.72–0.75). All scales demonstrated good reliability and validity. A structural equation model was established, and quantitative data and correlation analyses were conducted.

### 2.7 Data Collection

#### 2.7.1 General Situation Questionnaire

This questionnaire was developed by the researchers based on their research needs by integrating existing literature, expert consultations, and consideration of disease characteristics. It included items on age, sex, marital status, educational level, presence of children, duration of illness, adherence to a low‐copper diet, monthly income, source of income, type of medical insurance, and family history of WD.

#### 2.7.2 SCSQ

The SCSQ was developed by Zhang (2014) and comprises two dimensions: positive coping (12 items) and negative coping (8 items). It uses a 4‐point Likert scale, where 0 = “not adopted,” 1 = “occasionally adopted,” 2 = “sometimes adopted,” and 3 = “frequently adopted.” The score range for each dimension is 0–36 points for the positive coping dimension, 0–24 points for the negative coping dimension and the total score of the questionnaire is 0–60 points. The arithmetic average score of each dimension is obtained by dividing the sum of the item scores of each dimension by the number of items. The dimension with the highest mean score represented the manner in which patients responded. This questionnaire does not have a unified national cut‐off value. In clinical practice, the coping tendency is often judged by comparing the scores of different dimensions: if the score of the positive coping dimension is greater than that of the negative coping dimension, it is determined as “mainly positive coping.” Conversely, if the scores of the two dimensions are close (with a difference of <5 points), it is considered a “mixed response mode.” In the domestic test, the Cronbach's α of the two subscales was 0.89 and 0.78, respectively, and the content validity index ranged from 0.86 to 0.91. The questionnaire was self‐administered, with an average completion time of 3–5 min.

#### 2.7.3 SSRS

The SSRS developed by Anderson‐Butcher (Anderson‐Butcher et al. 2008) assesses social support using 10 items across three core dimensions: objective support (3 items), subjective support (4 items), and support utilization (3 items). The total score ranges from 12 to 66 points, with higher scores indicating higher levels of social support. The score ranges of each dimension are as follows: Objective support: 3–12 points (each item: 1–4 points); Subjective support: 4–28 points (each item: 1–7 points); Support utilization rate: 3–12 points (each item: 1–4 points). Scores <33 indicate low support, 33–45 indicate moderate support, and >46 indicate high support. The Cronbach's *α* was 0.90, indicating that the scale demonstrates excellent internal consistency and that the measurement results are stable and reliable. It was self‐administered, with an average completion time of 5–8 min.

#### 2.7.4 CDSMS

The Chinese version of the “Self‐Management Behavior Scale” in the CDSMS developed by Stanford University (Lin et al. 2013) is widely used in chronic disease self‐management research. It contains a total of 15 items, divided into 3 core dimensions. The specific dimensions and item distribution are as follows: movement management (6 items), cognitive symptom management (6 items), and communication with doctors (3 items). Each item is rated on a 5‐point scale (0–5 points). The total score ranges from 0 to 75 points, with higher scores indicating stronger self‐management ability. The scores for each dimension are as follows: 0–30 points for motor management, 0–30 points for cognitive symptom management, and 0–15 points for communication with physicians. Clinically, the CDSMS score is interpreted as follows: <45 points (<60%), low level of self‐management; 45–59.25 points (60%–79%), medium level of self‐management; ≥60 points (≥80%), high level of self‐management. It was self‐administered, with an average completion time of 4–6 min.

### 2.8 Statistical Analysis

Statistical analysis was performed using SPSS (v.23.0) and Amos (v.27.0). Quantitative data are expressed as mean ± standard deviation (x ± s), and descriptive variables are expressed as frequencies and constituent ratios. Correlation analysis was performed using Pearson's correlation coefficient. A significance level of *α* = 0.05 was adopted.

### 2.9 Model Fit

All fitting indicators of the model met the standard; the ratio of chi‐square to degrees of freedom (PCMIN/DF) was 2.410 < 3, and the comparative fit index (CFI) was 0.946 > 0.9.

## 3 Results

### 3.1 Participants

The general demographic and clinical characteristics of patients with WD are presented in Table 1.

**TABLE 1:** Characteristics of patients with Wilson disease.

Variable	Category	Frequency	Percentage
Sex	Male	139	60.4
	Female	91	39.6
Age	Under 20 years	87	37.8
	20 to 30 years old	78	33.9
	31–40 years old	47	20.4
	41–60 years old	17	7.4
	More than 60 years of age	1	0.4
Education	Junior high school and below	77	33.5
	Senior high school	58	25.2
	Junior college	37	16.1
	Regular college course	55	23.9
	Master's and above	3	1.3
Marriage	Spinster	157	68.3
	Married	55	23.9
	Divorced	16	7.0
	Widowed	2	0.9
Children	Have	62	27
	Do not have	168	73
Duration of sickness	0∼5 years	83	36.1
	5∼10 years	67	29.1
	10∼20 years	59	25.7
	>20 years	21	9.1
Adherence to low‐copper diet	Yes	200	97
	No	30	13
Monthly income	<1000 Yuan	128	55.7
	From 1000 to 3000 Yuan	36	15.7
	From 3000 to 5000 Yuan	26	11.3
	From 5000 to 7000 Yuan	23	10.0
	More than 7000 Yuan	17	7.4
Source of income	Agriculture	33	14.3
	Industry or project	41	17.8
	Business	9	3.9
	Others	147	63.9
Relatives with Wilson disease	Yes	55	23.9
	No	175	76.1

### 3.2 Scores of Coping Style, Social Support, and Self‐Management

Among the 230 patients with WD, the total coping style score was 50.44 ± 10.95, with mean positive and negative coping scores of 32.39 ± 8.16 and 18.06 ± 4.88, respectively. The total score for social support was 36.03 ± 7.71, which included scores for objective support (6.89 ± 2.4), subjective support (21.48 ± 4.88), and utilization of support (7.66 ± 2.51). The total score for self‐management behaviors was 37.76 ± 12.05, which included scores for exercise (13.6 ± 5.15), cognitive symptom management (15.31 ± 6.3), and communication with doctors (8.86 ± 3.84).

### 3.3 Relationship Between Self‐Management Behaviors, Coping Styles, and Social Support

Correlation analysis showed that the social support score was positively correlated with positive coping and self‐management behavior scores and negatively correlated with the negative coping score, while self‐management behavior was positively correlated with the positive coping score and negatively correlated with the negative coping score (Table 2).

**TABLE 2:** Correlation among social support, coping style, and self‐management in patients with Wilson disease (*r*‐value).

Variable	Social Support	Self‐management behaviors	Positive coping	Negative coping
Social support	1			
Self‐management behavior	0.386^**^	1		
Positive coping	0.578^**^	0.519^**^	1	
Negative coping	−0.170^**^	−0.308^**^	0.372^**^	1

** At the 0.01 level (two‐tailed), the association was significant.

### 3.4 Construction of Structural Equation Models for Coping Styles, Social Support, and Self‐Management

**FIGURE 1:**
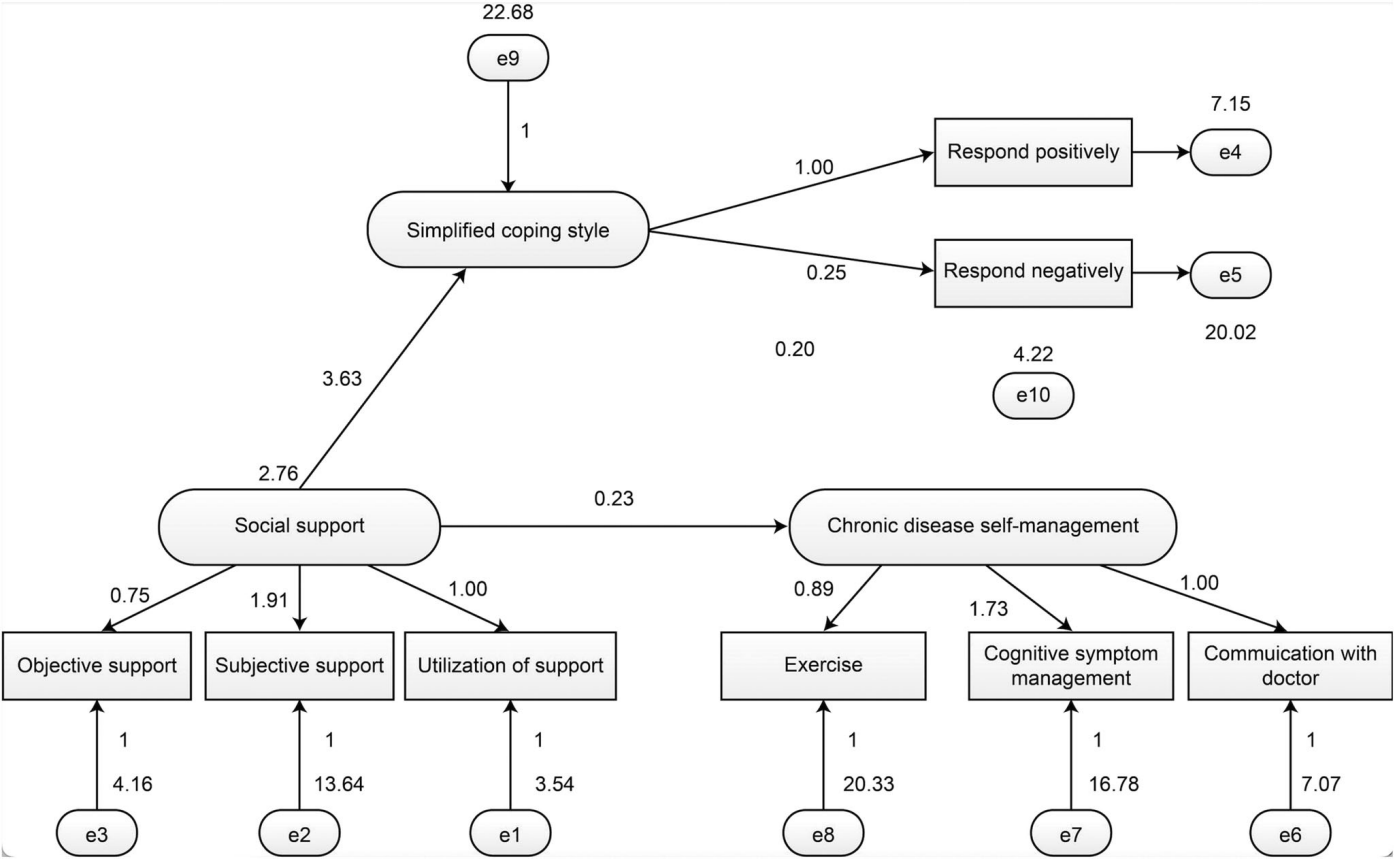
Structural equation model.

Based on prior research and the results of relevant analyses, a structural equation model was established, with social support as the exogenous latent variable and coping style and self‐management as the endogenous latent variables. The model is shown in Figure 1. The Tucker–Lewis Index (TLI) score was 0.910 (>0.9), and the root mean square error of approximation (RMSEA) was 0.078 (<0.08), indicating acceptable model fit. Social support exerted a direct effect on self‐management (*β* = 0.232, *p* < 0.001) and an indirect effect mediated by coping style. Coping style also exerted a direct effect on self‐management behavior (*β* = 0.199, *p* < 0.001). The direct and indirect effects of the variables are presented in Table 3, and the model path coefficients are listed in Table 4.

**TABLE 3:** Effect relationships between variables.

Model path	Direct effect	Indirect effect	Gross effect
Social support → Coping style	3.634		
Coping style → Self‐management behavior	0.199		
Social support → Self‐management behavior	0.232	0.723	0.955

**TABLE 4:** Path coefficients of the model.

Path	Estimate	SE	CR	P
Coping style ←‐ Social support	3.634	0.477	7.621	***
Self‐management behavior ←‐ Coping style	0.199	0.098	2.031	0.042
Self‐management behavior ←‐ Social support	0.232	0.402	0.577	0.564
Utilization of support ←‐ Social support	1.000			
Subjective support ←‐ Social support	1.908	0.259	7.361	***
Objective support ←‐ Social support	0.752	0.120	6.270	***
Positive coping style ←‐ Coping style	1.000			
Negative coping ←‐ Coping style	0.249	0.054	4.629	***
Communication with your doctor ←‐ Self‐management behavior	1.000			
Cognitive symptom management ←‐ Self‐management behavior	1.727	0.209	8.262	***
Exercise training ←‐Self‐management behavior	0.891	0.146	6.099	***

**Abbreviation**: CR, critical ration; SE, standard error.

## 4 Discussion

### 4.1 Low Level of Self‐Management Behavior in Patients With WD: A Manifestation of Maladjustment

Self‐management behavior refers to the patient's active efforts to maintain and promote their health; monitor and manage symptoms; reduce the physical, psychological, and social impact of the disease; and continue disease management under professional guidance (McCorkle et al. 2011). In this study, the total self‐management behavior score among 230 patients with WD was 37.76±12.05. Based on the classification threshold described above, the cut‐off value was changed to 50.3%. Consequently, self‐management behavior in this cohort was low (Li et al. 2023). This finding aligns with trends reported for other rare chronic diseases, where self‐management and treatment adherence are often suboptimal. For example, Göktaş and Yalcin (2024) reported substantial non‐adherence to WD medications in pediatric patients, underscoring that poor self‐management is a persistent challenge across populations with WD globally. Therefore, this finding is not an isolated phenomenon but rather reflects a broader pattern observed in rare chronic conditions requiring sustained self‐regulation.

From the perspective of Roy's adaptation model, self‐management is considered an adaptive response with the physiological and self‐concept modes (Zhang et al. 2021). Patients with WD must adhere to lifelong low‐copper diets and medication routines, which is an adaptive behavior in response to physiological stimuli. Our analysis of the causes of low levels of self‐management showed that most WD cases emerge during adolescence, a developmental stage characterized by unstable value systems. Consequently, patients may lack sufficient knowledge and motivation for disease control, contributing to inadequate health self‐management and poor adherence.

Furthermore, the low incidence of WD and the uneven distribution of medical resources further exacerbate the difficulty for patients to adapt to their role‐functional patterns. In the United States, Europe, and Asia, the clinical prevalence of WD is estimated to be 1/30,000 to 1/50,000 (Wungjiranirun et al., 2023). Many patients face barriers such as difficulty seeking medical treatment, limited health literacy, and a lack of rehabilitation support, all of which are external stimuli affecting their ability to fulfill family and social roles. According to Roy's model, maladaptation occurs when an individual cannot effectively respond to stimuli (Terela 2025). The low level of self‐management in patients with WD in this study was a manifestation of their insufficient overall adaptation. Therefore, long‐term involvement of healthcare professionals is crucial in supporting patients with WD to effectively improve their self‐management behaviors and overall adaptation.

### 4.2 A Study on the Correlation Between Social Support, Coping Styles, and Self‐Management Behaviors in WD Patients

This study showed that social support is positively correlated with self‐management behavior (*r* = 0.386) and also strongly positively correlated with positive coping styles (*r* = 0.578). This result can be interpreted in Roy's model as follows: social support functions as an important situational resource that helps patients acquire emotions and information by enhancing their interdependent mode. Good social support helps patients meet their demands for health resources and material support, enabling them to maintain a positive attitude and proactive behavior toward disease management. Bandura (2001) found that social support is an important component in regulating patients' self‐management behaviors. Warner et al. (2015) reported that patients receiving collaborative family support demonstrated greater motivation to engage in self‐management. These findings are consistent with our results, emphasizing the need for healthcare providers to encourage patients to actively utilize social support networks, reduce psychological pressure, and establish and maintain beneficial health behaviors, as well as encourage family members to care for and understand patients more emotionally.

Furthermore, we found that social support in patients with WD is positively correlated with positive coping and negatively correlated with negative coping. Positive coping styles reflect the adaptive strategies of patients in their self‐concept mode. Patients with stronger social support are more likely to adopt positive coping strategies, leading to improved disease management. Conversely, inadequate social support predisposes patients to adopt negative coping strategies, further weakening their self‐management ability. This is highly consistent with the logical chain of “stimulus—response—adaptation” in Roy's model.

The research also confirmed that the self‐management skills of WD patients are positively correlated with positive coping styles and negatively correlated with negative coping styles. A positive attitude toward illness may enhance the willingness of patients to actively manage the disease, acquire self‐management knowledge, improve their self‐management abilities, and effectively control disease progression, while maintaining attention to positive aspects of their lives. As WD requires long‐term pharmacological treatment and maintaining a low‐copper diet (Litwin et al. 2019), it may negatively affect the patient's psychology and physiology, which may lead to poor prognosis expectations and diminished confidence in self‐management. Studies have shown that patients with negative coping styles tend to experience loneliness, fear of illnesses, and emotional suppression (Zhang et al. 2023).

### 4.3 Coping Styles Mediate the Relationship Between Social Support and Self‐aManagement: Partial Adaptation Mechanism

The structural equation model showed that coping styles partially mediated the relationship between self‐management and social support. This indicates that social support directly promotes self‐management (*β* = 0.232, *p* < 0.001) and also indirectly enhances self‐management behaviors by improving positive coping styles (*β* = 0.199, *p* < 0.001). This indicates that higher levels of social support enable patients with WD to handle stressors more actively and manage their health behaviors more effectively. According to Roy's model, coping styles represent an individual's regulatory mechanisms when facing stimuli. Social support—as an external resource—optimizes this mechanism, enabling patients to achieve a better balance across multiple adaptive models such as physiology, self‐concept, and role function (Terela 2025). These findings suggest that healthcare professionals should actively involve family members in the long‐term management of patients with WD, enhance patients’ social support, and improve patients’ disease‐coping styles, thereby improving self‐management behaviors. In the clinical setting, nurses should provide professional social support to help patients standardize their self‐management behaviors.

### 4.4 Enhancing Social Support and Optimizing Coping Styles as Key Strategies for Improving Self‐Management in WD Patients

Guided by Roy's adaptation model and the results of this study, interventions for patients with WD should focus on “strengthening the social support system and promoting positive coping”—while accounting for unique characteristics of WD. For adolescent patients and those with limited access to medical resources, self‐management deficiencies can be addressed by establishing family support groups, connecting patients with public welfare economic assistance, and conducting online disease management courses (e.g., low‐copper diet education and symptom monitoring guidance). For patients with neurological disorders and cerebral WD, simplified coping skills training (such as visual charts for tracking symptoms and voice‐assisted communication tools) should be implemented to overcome communication and movement barriers and adopt positive coping methods more easily.

Furthermore, healthcare professionals should assume the role of “long‐term management guides,” track patients' access to social support and changes in their coping styles through regular follow‐ups, adjust intervention strategies in a timely manner, and promote patients' transformation from “low‐level self‐management” to “adaptive management.”

### 4.5 Strength and Limitations

By adopting a cross‐sectional survey design, this study was able to systematically obtain multi‐dimensional data on social support, coping styles, and self‐management within a relatively short period, enabling efficient exploration of association patterns among variables. Self‐management behavior in patients with WD plays a critical role in disease management. This study provided an initial assessment of the current status of self‐management behaviors in patients with WD and identified its influencing factors. However, the cross‐sectional design precludes definitive causal inference regarding the relationships among social support, coping style, and self‐management. Furthermore, the reliability of self‐reported data can be affected by the recall, response, and public acceptance biases. The generalizability of our findings may be limited by the single‐center design within the specific healthcare context of China. Given the centralized model of WD care and culturally influenced patterns of social support, our results should be validated in other populations with different healthcare systems (e.g., Europe or North America) and cultural norms.

### 4.6 Suggestions for Further Research

Building on these findings, future longitudinal studies are required to establish causality. Furthermore, the development and evaluation of structured interventions, such as coping skills training programs or family support–based initiatives, are needed to translate these correlational findings into tangible improvements in patients’ self‐management behaviors. At the same time, targeted intervention plans can be developed based on Roy's adaptation model, and the intervention effects can be tested through randomized controlled trials, providing more precise theoretical and practical support for the disease self‐management of Wilson's disease patients.

### 4.7 Summary of Findings Linked to Roy's Adaptation Model

Based on Roy's adaptation mode, the findings of this study can be explained through its four adaptive modes, reinforcing the model's applicability to patients with WD.

At the interdependence mode level, social support directly and positively influences self‐management behavior (r = 0.386) by satisfying patients' emotional, informational, and material needs (e.g., family participation and social network support) and strengthening positive coping (*r* = 0.578). This reflects the core logic of the model that “external resources promote individual adaptation.” Second, at the self‐concept mode level, coping styles serve as the core regulatory mechanism of this mode. Positive coping is positively associated with self‐management (*r* = 0.519) by enhancing patients’ disease cognition and treatment confidence (e.g., actively learning self‐management knowledge), whereas negative coping weakens self‐management (*r* = −0.308) by exacerbating negative emotions—reflecting the key role of internal cognitive–behavioral strategies in adaptive outcomes. Third, at the physiological mode level, patients with WD require long‐term adherence to a low‐copper diet and regular medication. The low self‐management score reflects patients’ inadequate adaptation to physiological stimuli (disease treatment needs) and confirms that self‐management is a critical adaptive output of the physiological mode. Fourth, at the role function mode level, the low incidence of WD (1/30,000–1/50,000) and uneven distribution of medical resources lead to difficulties in accessing medical care and impaired social role performance (e.g., limited family and social functions). The synergistic effect of social support and positive coping can alleviate this dilemma and promote patients’ role function adaptation. Furthermore, the partial mediating role of coping styles between social support and self‐management (*β* = 0.199, *p* < 0.001) further reveals the chain path of “external resources (interdependence) → internal regulation (self‐concept) → adaptive output (physiology/role function)” in the model, providing a clear theoretical foundation for clinically improving the multi‐dimensional adaptation level by strengthening social support and guiding positive coping.

## 5 Conclusion

Grounded in Roy's adaptation model, this study systematically explored the relationship between social support, coping styles, and self‐management behaviors of patients with WD. The findings revealed all three variables were at a moderate level and significantly interrelated. Coping styles played a partial mediating role between social support and self‐management, indicating that social support directly promotes self‐management and indirectly exerts its effect by enhancing positive coping. Our finding of suboptimal self‐management in Chinese patients with WD aligns with global challenges in the management of rare diseases, as reflected in the reported non‐adherence rates by Göktaş and Yalcin (2024). Although their pediatric study emphasized treatment knowledge, our study on adults revealed the critical psychosocial pathways through which social support enhances self‐management, both directly and indirectly, via positive coping styles. This underscores the need for comprehensive care that extends beyond knowledge dissemination to include strengthening social support and fostering adaptive coping strategies.

Roy's adaptation model provides a strong theoretical lens for interpreting these findings: (i) self‐management behaviors of patients with WD reflect their responses to multiple adaptation modes, shaped by external stimuli; and (ii) social support and coping styles are critical factors influencing their adaptation levels. The diagnosis and treatment of WD by healthcare professionals can enhance patients' social support and positive coping abilities, which in turn are posited to improve their self‐management behaviors and, ultimately, may contribute to an enhanced quality of life. This study provides theoretical guidance for the nursing practice of patients with WD, offers empirical evidence for improving their self‐management behaviors, and lays a foundation for future intervention research based on adaptive models.

## Author Contributions

**TingTing Zhan**: conceptualization, methodology, software, formal analysis, investigation, data curation, writing – original draft, and visualization. **Xiang Li**: conceptualization, methodology, validation, resources, writing – review and editing, supervision, project administration, and funding acquisition. **Yan Wang, Lili Wang, Caijie Sun, Xiaohan Hu**: investigation, resources, writing – review and editing. **Shutong Qiao**: software, formal analysis, and visualization. **Xue Li, Wenjie Tu**: formal analysis, project administration, and visualization.

## Funding

1.Strengthening the Constrcution of Major Difficult and Compliacted Diseases Integrated Traditional Chinese and Western Medicine Clinical Collaboration Project (Wilson's Disease),Grant No.ZDYN‐2024‐B‐013.2.2024 Anhui Provincial Department of Education Anhui University Scientific Research Project (Humanities Key Project):Construction and Clinical Application of Self‐Management Behavior Intervention System for Patients with Wilson's Disease,Grant No.2024AH052735.

## Ethics Statement

This study was approved by the Ethics Committee of the First Affiliated Hospital of Anhui University of Chinese Medicine (approval no.: 2021AH‐06; date: October 6, 2021).

## Consent

All patients provided written informed consent for participation.

## Conflicts of Interest

The authors declare no conflicts of interest.

## Data Source

Patients with Wilson's disease who visited a tertiary hospital in China from May 2022 to August 2022

## Reporting Method

This study follows the STROBE reporting guidelines. No patient or public involvement.

## Implications for Profession and/or Patient Care

This study supports nurses in developing interventions to improve self‐management behaviors in WD patients and helps identify factors influencing self‐management in this population.

## Data Availability

The datasets used and/or analyzed during the current study are available from the corresponding author upon reasonable request.
